# Genetic Characteristics and Pathogenicity Analysis in Chickens and Mice of Three H9N2 Avian Influenza Viruses

**DOI:** 10.3390/v11121127

**Published:** 2019-12-06

**Authors:** Yafen Song, Yong Zhang, Ling Chen, Bing Zhang, Min Zhang, Jingwen Wang, Ying Jiang, Chenghuai Yang, Taozhen Jiang

**Affiliations:** China Institute of Veterinary Drug Control, 8 Nandajie, Zhongguancun, Haidian District, Beijing 100081, China; songyf86527@163.com (Y.S.); zhangyong_1537@163.com (Y.Z.); chen2011521@163.com (L.C.); zhangbing06@163.com (B.Z.); zmbooksea@163.com (M.Z.); echowongbj@gmail.com (J.W.); jiangying_vet@163.com (Y.J.); ychenghuai@163.com (C.Y.)

**Keywords:** avian influenza virus, H9N2, phylogenetic analysis, pathogenicity, transmission, chicken, mice

## Abstract

H9N2 avian influenza is a remarkable disease that has circulated in domestic poultry in large regions of China and posed a serious threat to the poultry industry. The H9N2 virus can not only infect mammals directly, but also provide gene segments to generate novel, but lethal human reassortants. Therefore, it is important to study the evolution, pathogenicity, and transmission of the H9N2 virus. In this study, three H9N2 viruses isolated from chickens in different layer farms were identified. Phylogenetic analysis revealed that these H9N2 viruses were all multiple genotype reassortants, with genes originating from Y280-like, F/98-like, and G1-like viruses. Animal studies indicated that the AV1535 and AV1548 viruses replicated efficiently in the lungs, tracheas, spleens, kidneys, and brains of chickens; the viruses shed for at least 11 days post-inoculation (DPI) and were transmitted efficiently among contact chickens. The AV1534 virus replicated poorly in chickens, shed for 7 DPI, and were not transmitted efficiently among contact chickens. The AV1534 virus replicated well in mice lungs and caused about 2% weight loss. The AV1535 and AV1548 viruses were not able to replicate in the lungs of mice. Our results indicate that we should pay attention to H9N2 avian influenza virus surveillance in poultry and changes in the pathogenicity of them to mammals.

## 1. Introduction

H9N2 avian influenza virus (AIV), known as one of the predominant subtypes devastating the poultry industry, has circulated widely in the poultry population and caused huge economic losses. H9N2 avian influenza has been reported in all continents since the virus was first isolated from turkeys in North America in 1966 [1,2]. In Asia, H9N2 avian influenza viruses (AIVs) were only detected in apparently healthy ducks before the 1990s [3]. Subsequently, H9N2 viruses could be found in chickens in many Asian countries [4,5]. In China, the first outbreak of the H9N2 avian influenza was in chickens of Guangdong Province, from November 1992 to May 1994, and the virus was first identified in 1994 [4]. Afterwards, the H9N2 AIV rapidly spread to the majority of poultry-raising areas of China and 93.89% of chicken flocks were attacked by the viruses in the period 1996–2000 [6]. Now, the H9N2 AIVs are stable and established in domestic poultry in China.

Phylogenetic analysis reveals that the H9N2 AIVs derive from two major influenza gene pools: the North American lineage and the Eurasian lineage. The Eurasian lineage can be further divided into multiple virus sublineages, represented by A/chicken/Beijing/1/94 (BJ/94-like) or A/duck/Hong Kong/Y280/97 (Y280-like), A/quail/ Hong Kong/G1/97 (G1-like), A/chicken/Hong Kong/G9/97 (G9-like), A/duck/Hong Kong/Y439/97 (Y439-like), A/chicken/Shanghai/F/98 (SH/F98-like), etc. [6,7]. In China, G1-like viruses are predominantly found in quails in southern China, which were considered as the generators of the highly pathogenic H5N1 virus in 1997 [8]. BJ/94-like (Y280-like) and F/98-like viruses are preponderantly prevalent strains in chickens in the northern and eastern areas of China, respectively [9,10].

H9N2 AIVs can not only be isolated from different types of avian species, but also break through species barriers and directly infect mammalians without intermediate hosts. In 1998, H9N2 viruses were isolated from pigs and the first human-infecting H9N2 viruses were detected in five humans in China during the same period. Serological data revealed that there was an extremely high seropositive rate for H9N2 infections in humans who frequently come into contact with live birds. In addition, H9N2 viruses have also been detected in dogs, weasels, and mink [5]. More interestingly, H9N2 viruses not only infect mammals directly, but also provide partial or even a whole set of internal genes for emerging human-lethal H5N1, H7N9, H10N8, and H5N6 reassortants, posing a substantial threat to public health [6].

The capacity of the avian influenza virus to infect birds or humans is partially defined by the binding specificity of the hemagglutinin (HA), which is the major glycoprotein on the influenza virus surface. In general, the HA of human strains binds preferentially to α 2,6-linked sialic acid receptors, which are predominant in the respiratory epithelia in the upper respiratory tract of humans, whereas the HA of avian strains binds preferentially to α 2,3-linked sialic acid receptors which are abundant in the avian intestinal tract. A few amino acid changes in the HA protein can cause a switch from avian to human receptor specificity. Q226L and G228S substitution in the HA protein was sufficient for avian viruses to switch from α 2,3-linked sialic acid receptors to α 2,6-linked sialic acid receptors specificity [11]. In China, H9N2 influenza viruses with HA-Q226L substitution have increasingly been found. HA-226L allows H9N2 viruses to preferentially infect cells expressing mainly α 2,6-linked sialic acid receptors and grow more efficiently in human airway epithelia cells [12].

Therefore, the continuous isolation of H9N2 viruses from poultry worldwide, along with their ability to infect mammals and reassort with other influenza viruses, makes it important to understand the evolution and properties of these viruses. In this study, we provide a preliminary characterization of the genetic and biological properties of three H9N2 viruses isolated from different layer farms in Shandong Province of East China in 2011.

## 2. Materials and Methods

### 2.1. Virus Isolation

The three H9N2 viruses—(A/chicken/Shandong/AV1534/2011(H9N2) (AV1534), A/chicken/Shandong/AV1535/2011(H9N2) (AV1535), and A/chicken/Shandong/AV1548/2011(H9N2) (AV1548)—were isolated from chickens in different layer farms in Shandong Province of eastern China in 2011. The virus subtype was identified by a standard hemagglutination inhibition (HI) test, reverse-transcription polymerase-chain reaction (RT-PCR), and real-time RT-PCR [13,14]. In brief, fecal samples were treated with phosphate-buffered saline (PBS) containing 2000 units/mL penicillin and 2000 units/mL streptomycin, and they were inoculated into 9- to 10-day-old specific-pathogen-free (SPF) embryonated chicken eggs though allantoic and amniotic routes. The HA-positive allantoic fluid was harvested after incubation at 37 °C for 72 h. The HA subtype was identified by the HI test and specific anti-sera was obtained from Harbin Veterinary Research Institute, China. The NA subtype was accomplished using real-time RT-PCR. Furthermore, these viruses were confirmed again as H9N2 viruses by nucleotide sequence and a BLAST search of the Influenza Sequence Database in GenBank. The viruses were subsequently passaged three times with the inoculation of 9- to 10-day-old SPF embryonated chicken eggs by limiting dilution assay before being stored at −70 °C. Values of 50% embryo infective doses (EID_50_) were calculated by the Reed–Muench method [15].

### 2.2. Phylogenetic and Molecular Analysis

RNA extraction and cDNA synthesis were carried out as described previously [16]. The PCR products were purified with the QIAquick PCR purification kit (Qiagen, Hilden, Germany) and sequencing was performed by Shanghai Invitrogen Biotechnology Co., Ltd. The sequence fragments were assembled, and residue analyses were performed both using Lasergene 7.1 (DNASTAR, Madison, WI, USA). Eight phylogenetic trees of the three H9N2 influenza viruses were generated by the distance-based neighbor-joining method, using software MEGA 4.0 (Sinauer Associates, Inc., Sunderland, MA, USA). The reliability of the trees was assessed by bootstrap analysis with 1000 replicates. Horizontal distances were proportional to genetic distance. The nucleotide sequences are available from GenBank under the accession numbers: AV1534: MN686364-MN686371; AV1535: MN686272-MN686279; AV1548: MN686255-MN686262.

### 2.3. Animal Studies

#### 2.3.1. Chickens

Six 6-week-old white leghorn SPF chickens in each group were intranasally inoculated with 10^6^ EID_50_ of the AV1534, AV1535, and AV1548 viruses in 0.2 mL, respectively. At 24 h post-infection, three chickens were inoculated intranasally with 0.2 mL PBS as a contact group housed with those chickens inoculated with AV1534, AV1535, and AV1548 viruses, respectively. All chickens were observed for clinical signs for 14 days. Oropharyngeal and cloacal swabs were collected on 1 day post-inoculation (DPI), 3, 5, 7, 9, 11, and 13 DPI. In order to detect the virus replication, three infected chickens in each group were killed on 3 DPI and lungs, brain, spleen, kidneys, and trachea were collected. All swabs and tissues were titrated for virus titers in eggs. Sera was collected 14 DPI for seroconversion tests.

#### 2.3.2. Mice

Sixty-eight 6- to 8-week-old SPF female BALB/c mice were divided into four groups. Seventeen mice were anesthetized with CO_2_ and inoculated intranasally with 10^6^ EID_50_ of the three H9N2 influenza viruses in a volume of 50 μL. Seventeen mice were inoculated with PBS as the control. Three mice in each group were euthanized 1, 2, 3, and 5 DPI, and their lungs, brain, spleen, and kidneys were collected for virus titration in eggs. The remaining five mice were monitored daily for weight loss and mortality for 14 days.

### 2.4. Ethics Statements

Six- to 8-week-old SPF female BALB/c mice were purchased from Beijing Vital River Laboratory Animal Technology Co., Ltd., China. The 6-week-old white leghorn SPF chickens and 9–10-day-old specific-pathogen-free (SPF) embryonated chicken eggs were purchased from Beijing Boehringer Ingelheim Vital Biotechnology Co., Ltd., China. All animal experiments were done in biosafety level 2+ facilities and were conducted in accordance with guidelines of animal welfare of applicable laws and guidelines and were approved by the Ethics Committee of the China Institute of Veterinary Drug Control (201800177; 19 December 2018).

## 3. Results

### 3.1. Phylogenetic Analysis of the Three H9N2 Viruses

To understand the genetic origins and molecular evolution of the three H9N2 viruses, three H9N2 viruses isolated from different layer farms in Shandong were completely sequenced. All eight gene segments of these viruses were characterized and phylogenetically analyzed. The sequence data of the H9N2 viruses and other available sequence data were all obtained from the NCBI Influenza Virus Resource.

**Figure 1:**
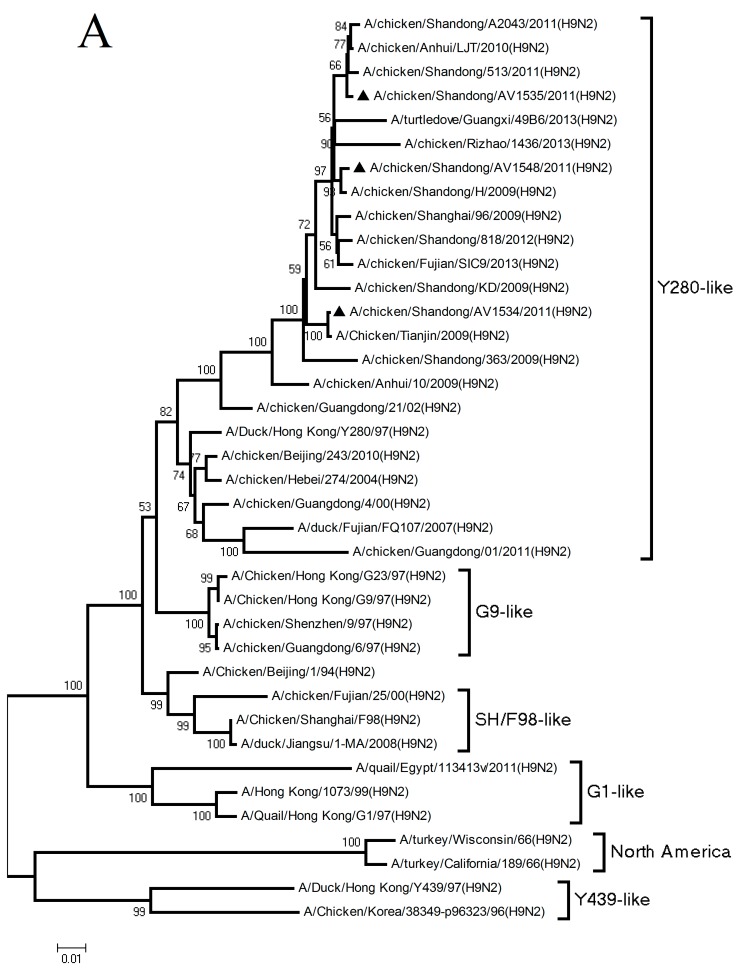

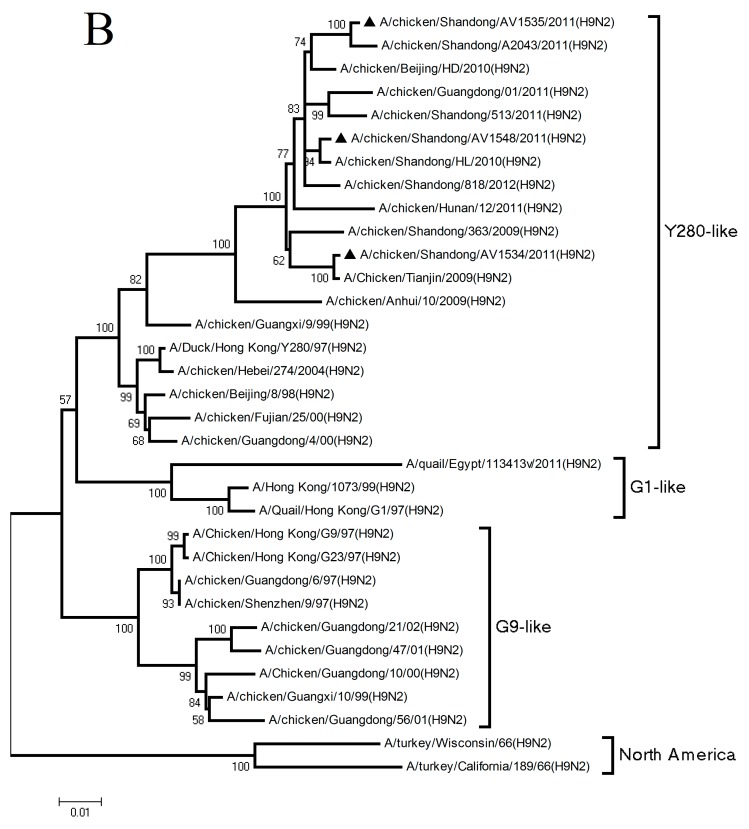

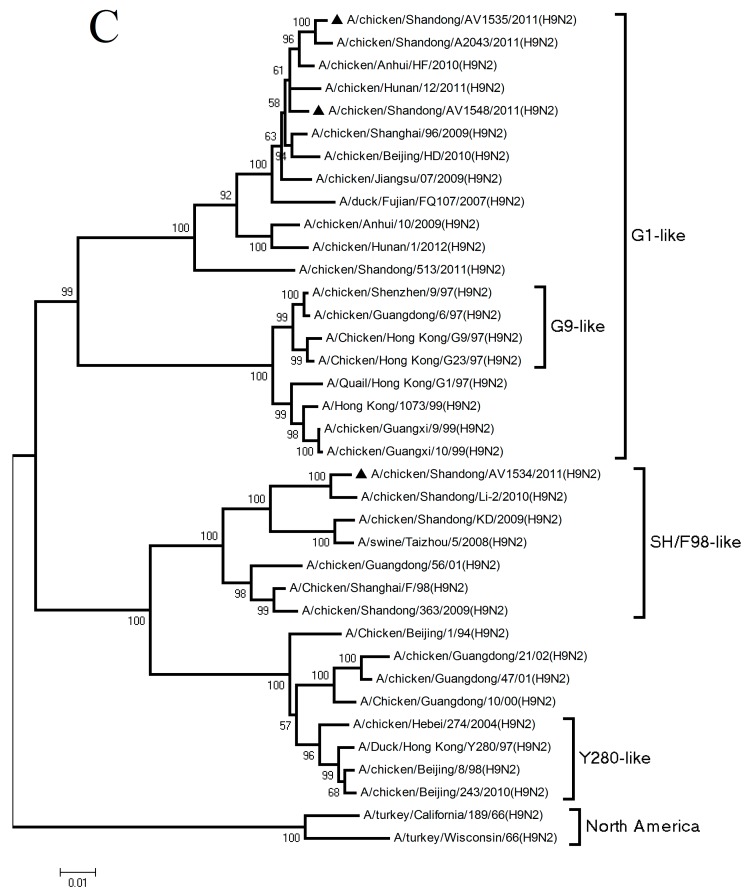

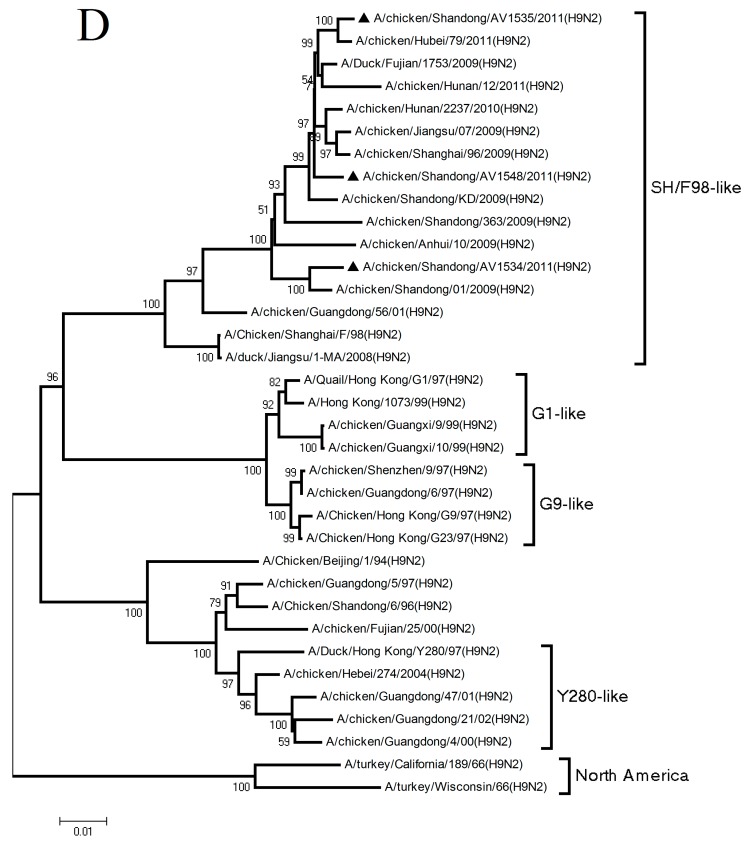

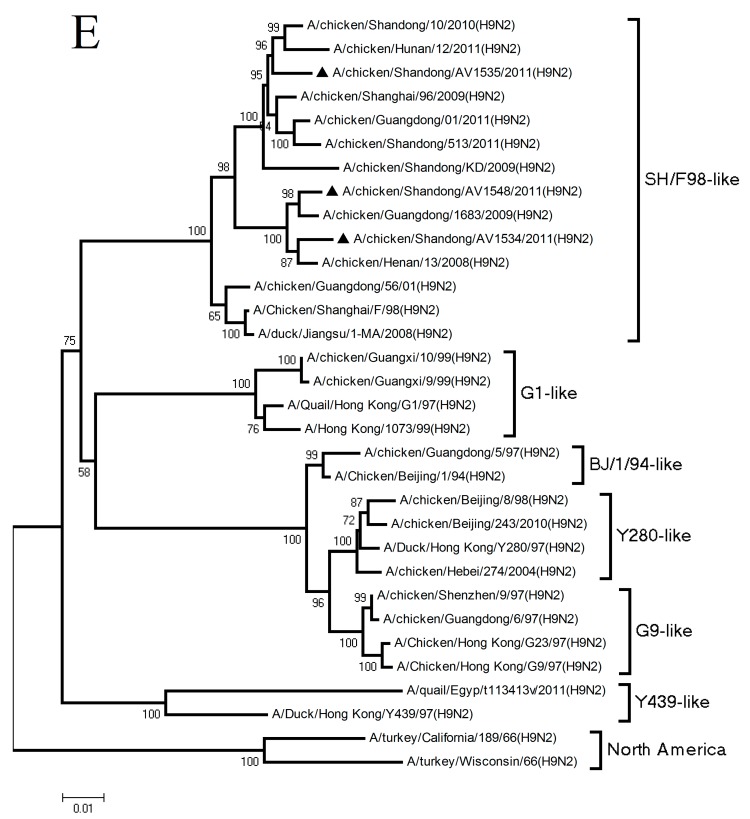

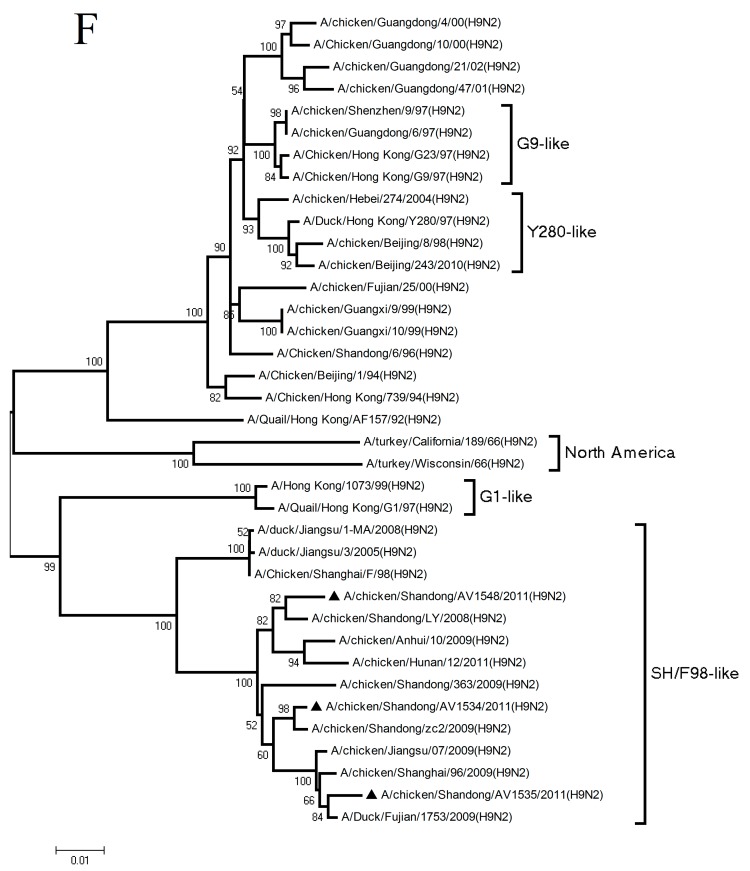

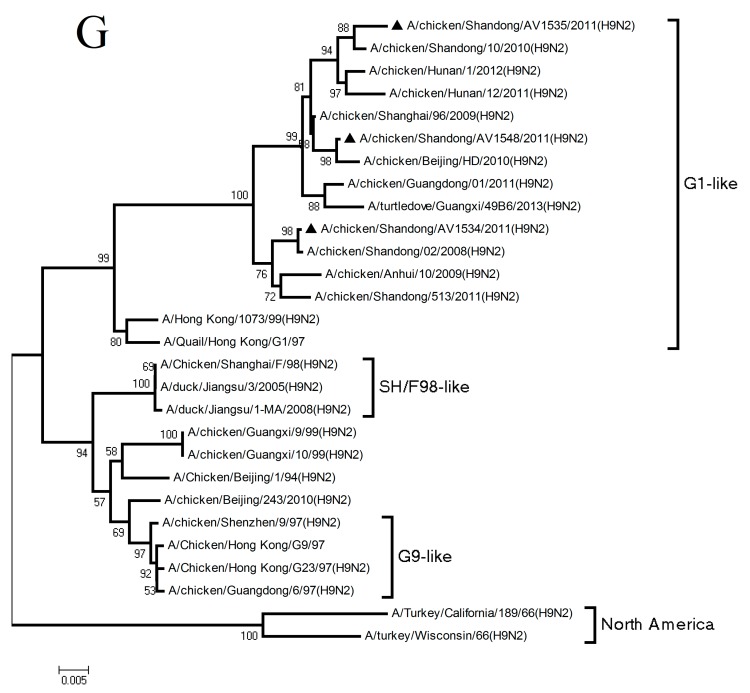

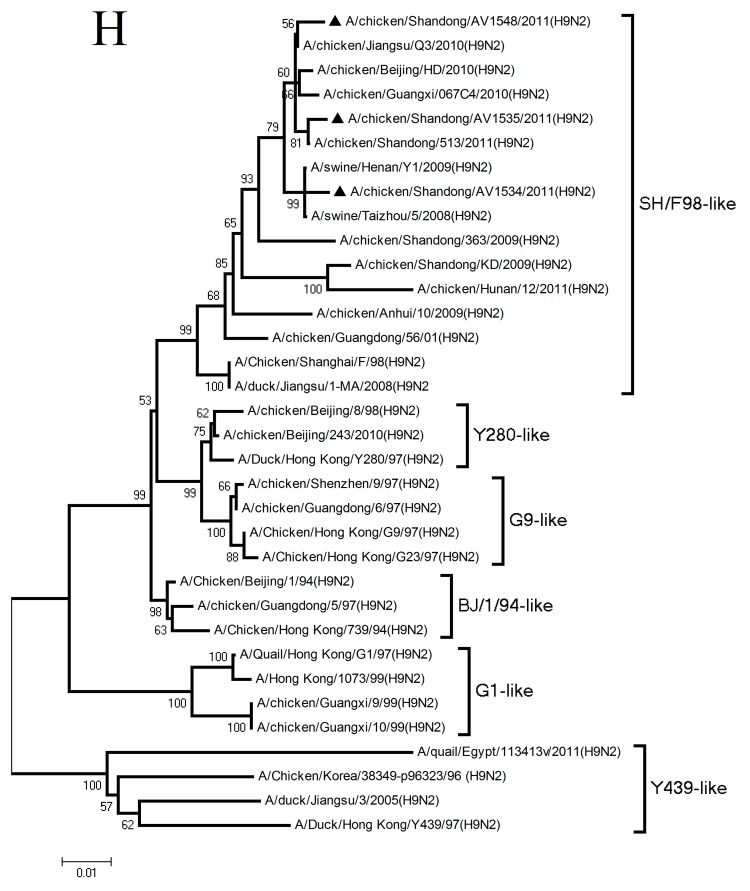
Phylogenetic trees of the (**A**) hemagglutinin (HA), (**B**) neuraminidase (NA), (**C**) polymerase basic subunit 2 (PB2), (**D**) polymerase basic subunit 1 (PB1), (**E**) polymerase acidic subunit (PA), (**F**) nucleoprotein (NP), (**G**) matrix (M), and (**H**) nonstructural (NS) genes of H9N2 influenza viruses. The trees were generated by the neighbor-joining method of the MEGA 4.0 with 1000 bootstrap replicates. The isolated viruses in this study were marked with a black triangle “▲”.

**Figure 2:**
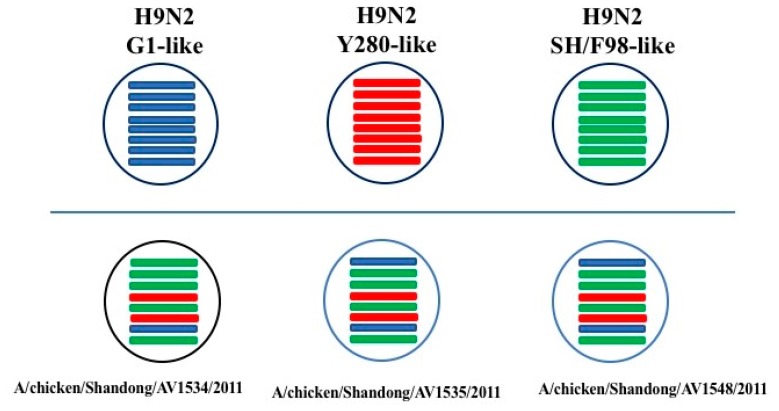
Genetic reassortants of the three H9N2 avian influenza viruses. The eight gene segments of the three H9N2 viruses, represented by horizontal bars are, from top to bottom, PB2, PB1, PA, HA, NP, NA, M, and NS. Each different color represents a distinct origin.

Sequence analysis demonstrated that the three H9N2 viruses were 85.7–99.5% and 93.9–99.5% homologous, with another at the nucleotide and amino acid levels, respectively. Phylogenetic analysis of the HA genes revealed that AV1534, AV1535, and AV1548 viruses all fell into the Y280-like lineage (Figure 1A). The HA gene of AV1534 virus was closely related to A/Chicken/Tianjin/2009(H9N2), with a nucleotide homology of 97.9%. The HA gene of AV1535 virus was 99.1% homology with A/chicken/Anhui/LJT/2010(H9N2). The HA gene of AV1548 virus had 99.6% homology compared with A/chicken/Shandong/H/2009(H9N2) (Figure 2 and Table 1).

The NA genes of the three H9N2 viruses were also clustered into the Y280-like lineage (Figure 1B). The NA genes of AV1534, AV1535, and AV1548 viruses were 99.8%, 98.3%, and 99.4% homologous with A/Chicken/Tianjin/2009(H9N2), A/chicken/Beijing/HD/2010(H9N2), and A/chicken/Shandong/HL/2010(H9N2), respectively (Figure 2 and Table 1).

Phylogenetic analysis of the six internal genes demonstrated that the polymerase acidic subunit 2 (PB2) genes of the AV1535 and AV1548 viruses both belonged to the G1-like lineage and were 98.9% and 99.1% homologous with A/chicken/Anhui/HF/2010(H9N2) and A/chicken/Anhui/HF/2010(H9N2), respectively. The PB2 gene of the AV1534 virus fell into the SH/F98-like lineage and had 98.7% homology with A/chicken/Shandong/Li-2/2010(H9N2) (Figure 1C and Table 1). The polymerase acidic subunit 1 (PB1), polymerase acidic subunit (PA), nucleoprotein (NP), and nonstructural (NS) genes of the three H9N2 viruses were all incorporated into the SH/F98-like lineage (Figure 1D–F,H). The PB1 genes of AV1534, AV1535, and AV1548 viruses were 98.9%, 99.2% and 99.1% homologous with A/chicken/Shandong/01/2009(H9N2), A/Duck/Fujian/1753/2009(H9N2), and A/chicken/Hunan/2237/2010(H9N2), respectively (Figure 2 and Table 1). The PA genes of the three viruses were 98.8%, 98.5%, and 99.1% homologous with A/chicken/Henan/13/2008(H9N2), A/chicken/Shanghai/96/2009(H9N2), and A/chicken/Guangdong/1683/2009(H9N2), respectively (Figure 2 and Table 1). The NP gene of AV1534, AV1535, and AV1548 viruses were closely related to A/chicken/Shandong/zc2/2009(H9N2), A/Duck/Fujian/1753/2009(H9N2), and A/chicken/Shandong/LY/2008(H9N2), with homologies of 99.3%, 98.9%, and 98.6%, respectively (Figure 2 and Table 1). The NS genes of AV1534, AV1535, and AV1548 viruses were 99.5%, 99.4%, and 99.5% homologous, compared with A/swine/Taizhou/5/2008(H9N2), A/chicken/Jiangsu/Q3/2010(H9N2), and A/chicken/Jiangsu/Q3/2010(H9N2), respectively (Figure 2 and Table 1).

Phylogenetic analysis of the matrix (M) genes showed that all of the three viruses were clustered into the G1-like lineage (Figure 1G). The M gene of the AV1534 virus was closely related to the A/chicken/Shandong/02/2008(H9N2), with a homology of 99.9% (Figure 2 and Table 1). The M gene of the AV1535 virus was 99.3% homologous with A/chicken/Shandong/10/2010(H9N2). The M gene of the AV1548 virus was both closely related to A/chicken/Beijing/HD/2010(H9N2) and A/chicken/Shanghai/96/2009(H9N2), with a homology of 99.6% (Figure 2 and Table 1).

Taken together, the three H9N2 viruses in this study showed a reassortment among different H9N2 viral genotypes. All of their surface genes (HA and NA) were derived from the Y280-like lineage. Their internal genes (PB2, PB1, PA, NP, M, and NS) were originated from either the SH/F98-like or G1-like lineages.

### 3.2. Molecular Characterization

**Figure 3:**
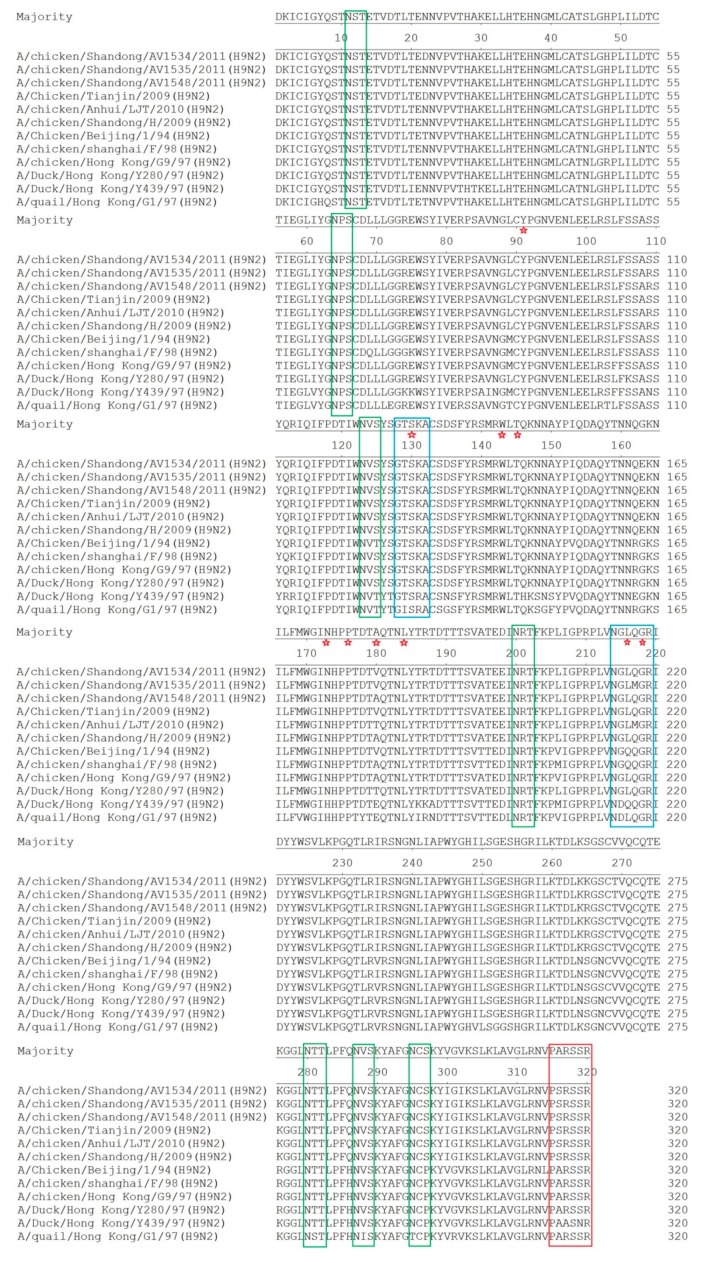
Molecular analysis of the HA1 amino acid sequences of the three H9N2 avian influenza viruses and reference viruses. Potential N-glycosylation sites (PGS) were marked with a green shade. Pentagram indicates the previously defined receptor-binding sites (RBS). The right edge of the binding pocket (128–132) and the left edge of the binding pocket (214–219) are marked with a blue shade. A red shade represents the cleavage site.

To understand the molecular basis of the pathogenicity and interspecies transmission of these H9N2 viruses, the deduced amino acid sequences of viral proteins were analyzed and compared with those of other H9N2 reference viruses. As shown in Figure 3, all of these three H9N2 viruses possessed the same amino acid sequence of PSRSSR/G at the cleavage site between HA1 and HA2, representing low pathogenicity in chickens. Compared to the H9N2 reference viruses, six potential glycosylation sites (PGS) (N–X–S/T) were conserved at positions 11(NST), 64(NPS), 123(NVS), 200(NRT), 280(NTT), and 287(NVS) in the HA1 protein of the AV1534, AV1535, and AV1548 viruses. Interestingly, the amino acid P to S change at position 297 led to the addition of one potential PGS in the AV1534, AV1535, and AV1548 viruses, which has been found in the HA1 protein of the recent H9N2 viruses isolated from chickens [17,18,19]. Remarkably, all of the three H9N2 viruses had a human-like motif 216-Leu (H9 numbering) at the receptor binding site. Compared to A/duck/Hong Kong/Y280/97, T180V (A) mutation could be found in the receptor-binding site of the three H9N2 viruses. Different from the AV1534 and AV1548 viruses, there was an Q217M mutation in the left-edge of the binding pocket of the AV1535 virus.

All of the three H9N2 viruses encode a deletion at positions 63–65 (N2 numbering) of the NA stalk region, which resulted in the loss of one potential PGS. These H9N2 viruses did not have E627K and D701N mutations in the PB2 protein that are associated with increased virulence of influenza viruses [20,21,22]. However, the AV1534 virus had an A588I substitution in the PB2 protein, which could enhance virus replication in mammalian cells and virulence in mice [23]. All of the three H9N2 viruses had an S31N mutation in the M2 protein, which is associated with amantadine resistance [24]. The PDZ domain ligand (PDZ-L) of full-length avian NS1 comprised residues 227–230 aa and could increase the virulence of AIVs in humans [25]. The deduced amino acid sequences of the NS1 genes of the three H9N2 viruses were 217 aa in length and lacked the C-terminal amino acids, which contain a PDZ domain ligand (PDZ-L). The three H9N2 viruses had the NS1 S42 mutation that plays an important role in the inhibition of IFN production and in the virulence of the virus in mice [26].

### 3.3. Pathogenicity and Transmission of These H9N2 Viruses in Chickens

To understand the pathogenicity and transmission of H9N2 viruses in chickens, 6-week-old white leghorn SPF chickens were inoculated intranasally with 0.2 mL 10^6.0^ EID_50_ of the AV1534, AV1535, and AV1548 viruses, respectively. At 24 h post-infection, three chickens were inoculated intranasally with 0.2ml PBS as a contact group housed with those chickens inoculated with viruses. All chickens were observed for 14 days for clinical signs and deaths.

None of the three H9N2 viruses induced clinical signs or deaths in chickens during the observation period. All of the inoculated and contact chickens seroconverted at 14 DPI.

To detect the virus replication in the organs of inoculated chickens, lungs, trachea, spleen, kidneys, and brais were collected at 3 DPI. The results showed that the AV1534 virus replicated to mean titers of 2.58 log_10_EID_50_/0.1 mL, 2.17 log_10_EID_50_/0.1 mL, 1.67 log_10_EID_50_/0.1 mL, and 1.92 log_10_EID_50_/0.1 mL in the lungs, trachea, spleen, and brain, respectively. AV1535 and AV1548 viruses could be detected from all the organs tested, and their mean titers were 4.75–5.42 log_10_EID_50_/0.1 mL, 3.50–3.50 log_10_EID_50_/0.1 mL, 2.83–2.17 log_10_EID_50_/0.1 mL, 2.25–2.83 log_10_EID_50_/0.1 mL, and 2.25–1.75 log_10_EID_50_/0.1 mL in the lungs, trachea, spleen, kidneys, and brain, respectively (Table 2).

To measure the virus shedding, oropharyngeal and cloacal swabs of the chickens inoculated with the AV1534, AV1535, and AV1548 viruses were obtained 1, 3, 5, 7, 9, 11, and 13 DPI (Table 3). The virus shedding of the AV1534-inoculated group was detected from oropharyngeal swabs of all the inoculated chickens at 1, 3, 5, and 7 DPI, and virus titers were from 2.75 log_10_EID_50_/0.1 mL to 4.42 log_10_EID_50_/0.1 mL. The virus shedding was detected from cloacal swabs of 3/6 to 6/6 chickens from 1 to 7 DPI, and virus titers were from 1.83 log_10_EID_50_/0.1 mL to 2.71 log_10_EID_50_/0.1 mL. From 9 to 13 DPI, the virus shedding of the AV1534-inoculated group could not be detected from oropharyngeal and cloacal swabs. In the AV1535-inoculated group, virus shedding was detected from the oropharyngeal swabs of 67–100% of chickens at 1, 3, 5, and 7 DPI and virus titers were from 2.75 log_10_EID_50_/0.1 mL to 3.83 log_10_EID_50_/0.1 mL. Virus shedding was detected from the cloacal swabs of 17–100% chickens from 1 to 13 DPI and virus titers were from 1.63 log_10_EID_50_/0.1 mL to 2.83 log_10_EID_50_/0.1 mL. In the AV1548-inoculated group, the virus shedding was detected from oropharyngeal swabs of all the inoculated chickens at 1, 3, 5, and 7 DPI, and virus titers were from 2.42 log_10_EID_50_/0.1 mL to 4.50 log_10_EID_50_/0.1 mL. The virus shedding could not be detected from the oropharyngeal swabs at 9, 11, and 13 DPI. Virus shedding was detected from the cloacal swabs of 33–67% chickens from 1 to 11 DPI and virus titers were from 1.58 log_10_EID_50_/0.1 mL to 2.33 log_10_EID_50_/0.1 mL. The virus shedding could not be detected from the cloacal swabs at 13 DPI.

To investigate the transmissibility of the three H9N2 viruses in chickens, oropharyngeal and cloacal swabs of the contact chickens were also obtained 1, 3, 5, 7, 9, 11, and 13 DPI (Table 3). For the AV1534-contacted chickens, the virus shedding was detected in the oropharyngeal swabs of 100% of the chickens from 1 to 5 DPI and virus titers were from 3.08 log_10_EID_50_/0.1 mL to 4.17 log_10_EID_50_/0.1 mL. At 7 DPI, only one chicken was detected virus shedding from the oropharyngeal swabs. The virus could not be detected from the oropharyngeal swabs at 9, 11, and 13 DPI. The virus was detected in 1/3 of chickens from the cloacal swabs at 3 and 5 DPI, and virus titers were both 1.83 log_10_EID_50_/0.1 mL. The virus could not be detected from the cloacal swabs at 1, 7, 9, 11, and 13 DPI. For the AV1535-contacted chickens, the virus shedding was tested from both oropharyngeal and cloacal swabs at 1, 3, 5, and 7 DPI. The virus titers were from 2.25 log_10_EID_50_/0.1 mL to 3.25 log_10_EID_50_/0.1 mL in oropharyngeal swabs and 1.58 log_10_EID_50_/0.1 mL to 2.33 log_10_EID_50_/0.1 mL. Remarkably, from 9 to 13 DPI, the virus shedding could also be detected from cloacal swabs. For the AV1548-contacted chickens, the virus shedding could be detected from the oropharyngeal swabs of all chickens within 11 DPI and the virus titers were from 1.75 log_10_EID_50_/0.1 mL to 4.08 log_10_EID_50_/0.1 mL. Virus shedding was detected from cloacal swabs of 33–100% of the contacted-chickens at 1 to 7 DPI and the virus titers were from 1.92 log_10_EID_50_/0.1 mL to 2.50 log_10_EID_50_/0.1 mL.

In sum, the results indicated that, except for the AV1534 virus, these H9N2 viruses could replicate efficiently in multiple organs of inoculated chickens, and all of these viruses were generally shed in higher titers from the respiratory tract than the intestinal tract. However, the virus shedding of these viruses in cloacal swabs could last for a longer time than in oropharyngeal swabs. Therefore, our results suggest that these H9N2 viruses could be transmitted efficiently among contact chickens via the respiratory tract and intestinal tract.

### 3.4. Pathogenicity of These H9N2 Viruses in Mice

**Figure 4:**
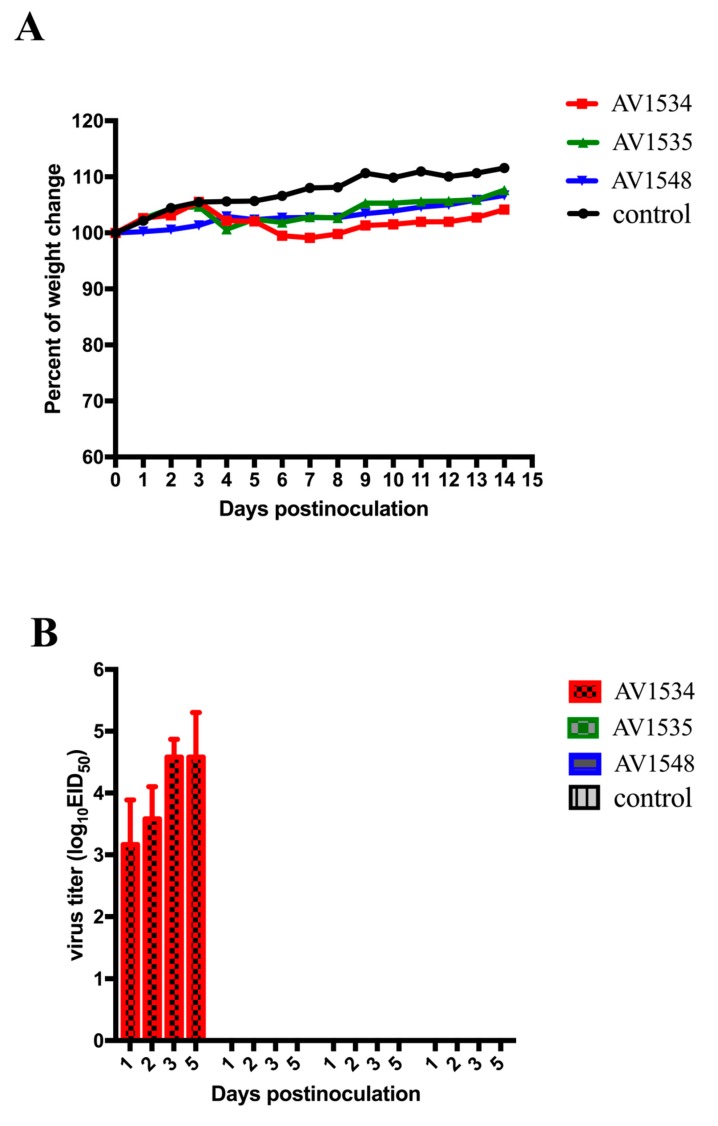
Replication of the three H9N2 avian influenza viruses in mice. (**A**) Weight change of BABL/c mouse during the 14 DPI. (**B**) Six-week-old SPF BALB/c mice were inoculated intranasally with 10^6^EID_50_ of each virus in a 50 μL volume. Three mice were killed on 1, 2, 3, and 5 DPI, respectively. Lungs were collected for virus titration in eggs. A value of 1.0 was assigned for calculations if the virus was not detected from the undiluted sample in three embryonated chicken eggs. The virus titers were shown as mean ± SD.

To detect whether these H9N2 AIVs could cross the interspecies barrier and infect mammalian hosts, 68 6- to 8-week-old female BALB/c mice were inoculated intranasally with 10^6^EID_50_ of the three H9N2 influenza viruses in a volume of 50 μL, respectively. Organs from three mice in each group were collected on 1, 2, 3 I, and 5 DPI. The remaining five mice were monitored daily for weight loss and mortality for 14 days. Our results found that the AV1534 virus could replicate well in the lungs with titers of 3.17–4.58 log_10_EID_50_/0.1 mL of inoculated mice on 1, 2, 3, and 5 DPI and the weight loss of mice was approximately 2% over 8 DPI. The AV1535 and AV1548 viruses were not recovered from any of the organs of the inoculated mice on 1, 2, 3, and 5 DPI, and the mice continued to gain weight during the observation period. None of the viruses were detected from the spleen, kidneys, and brain, and no deaths were found during the experiment (Figure 4A,B).

In summary, these H9N2 AIVs had different abilities to infect mice, and these H9N2 AIVs might have the ability to infect mammals and even humans.

## 4. Discussion

The H9N2 avian influenza virus, considered a low pathogenic virus, has been circulating worldwide and isolated from different types of terrestrial poultry [27]. Direct infection or co-infection with other pathogens often result in mild-to-severe respiratory disease signs, decreases in egg production, high mortality, and great economic losses in the poultry industry [28]. As a low-pathogenicity pathogen, the H9N2 virus is more likely to infect and transmit asymptomatically among birds, which could increase the chance of gene reassortment with other AIVs during co-infection. Therefore, it is important to know the genetic evolution, viral pathogenicity, and transmissibility of the H9N2 AIV.

In this study, we described the genetic and biologic properties of three H9N2 viruses isolated from chickens in different layer farms in Shandong Province of eastern China in 2011. The results demonstrated that AV1534, AV1535, and AV1548 viruses had reassorted among different H9N2 viral genotypes. All of their surface genes (HA and NA) were derived from the Y280-like lineage, while their internal genes (PB2, PB1, PA, NP, M, and NS) were originated from either the SH/F98-like or G1-like lineages.

In China, although large-scale farms have gradually become the main feeding and management models, traditional small-scale and backyard-level raisings, many with chickens and ducks, as well as pigs, in close proximity, still occupy a certain ratio of poultry production nationwide. In these feeding models, biosecurity condition and vaccination coverage are impossible to implement and these backyard flocks usually access poor veterinary services, which might lead to nonrecognition or delayed recognition of disease outbreaks. Once these asymptomatic but virus-bearing poultry infect other influenza A viruses, reassortment events will occur. If this does occur, it facilitates the rapid generation of novel influenza A viruses, potentially including antigenic and drug resistance variants. In our study, it was noteworthy that the PB1 and NP genes of the AV1535 virus were closely related to A/duck/Fujian/1753/2009 (H9N2), which was isolated from ducks in live bird markets in 2009 [29]. The NS gene of the AV1534 virus was closely related to A/swine/Taizhou/5/2008 (H9N2), isolated from swine in 2008. These results supported the idea of a two-way transmission of influenza A virus between terrestrial and aquatic birds [30], and pigs serve as potential “mixing vessels” for avian, swine, and human influenza viruses [31].

The pathogenesis of avian influenza virus is complex and the ability of these viruses to produce disease and death in avian species is usually dependent on viral, host, and environmental factors. The HA protein, the major viral surface glycoprotein, is responsible for the binding of the virus particle to cell surface receptors of the host cell. Receptor specificity and proteolytic activation of fusion capacity are major determinants of tissue tropism and virus dissemination in the organism. Low pathogenic avian influenza (LPAI) viruses require trypsin-like proteases to cleave their HA protein. These enzymes are usually available in the mucosal epithelial cells of the respiratory and intestinal tracts, which limit the tissue distribution of these viruses. In contrast, highly pathogenic avian influenza (HPAI) viruses have multiple basic amino acids at the cleavage site of the HA protein. This allows the HA of HPAI viruses to be cleaved by furin-like proteases present in many organs, which results in systemic infection and viruses can be isolated from many organs. This suggests that systemic infection could be a determinant of virulence of avian influenza virus to host. Previous studies demonstrated that some early BJ/1/94-like viruses induced mortality rates as high as 80% in infected birds, and the disease signs caused by BJ/1/94-like viruses were confined to the lungs and could not be systemically spread [1]. However, most of the H9N2 viruses were not lethal for chickens, and these studies only detect the ability of these viruses to replicate in the respiratory tract and intestinal tract of chickens. It is still unclear whether these viruses could replicate in other organs [1,9,27,32,33,34]. In the present study, we demonstrated that all of these viruses were generally shed in higher titers from the respiratory tract and the intestinal tract. Also, the virus shedding of these viruses in cloacal swabs could last for a longer time than in oropharyngeal swabs. Our results also suggest that these H9N2 viruses could be transmitted efficiently among contact chickens via the respiratory tract and intestinal tract. It was noteworthy that AV1535 and AV1548 viruses not only replicated efficiently in the lungs and trachea (their mean titers were 4.75–5.42 log_10_EID_50_/0.1 mL and 3.50–3.50 log_10_EID_50_/0.1 mL), but also were detected in the spleen, kidneys, and brain (their mean titers were 2.83–2.17 log_10_EID_50_/0.1 mL, 2.25–2.83 log_10_EID_50_/0.1 mL, and 2.25–1.75 log_10_EID_50_/0.1 mL, respectively). Although AV1534 virus was not be detected in the kidneys, it replicated in the lungs, trachea, spleen, and brain (their mean titers were 2.58 log_10_EID_50_/0.1 mL, 2.17 log_10_EID_50_/0.1 mL, 1.67 log_10_EID_50_/0.1 mL, and 1.92 log_10_EID_50_/0.1 mL, respectively). Previous studies have shown that the nucleotide sequences of the HAs of the H9 viruses had two different structural motifs at the carboxyl terminus of HA1: one motif was X–X–X–R (where X and R represent non-basic and basic amino acids, respectively), representing the nonpathogenic avian influenza viruses; the other was R-S-S-R, similar to the motif (R–X–R/K–R), which is required for highly pathogenic viruses of the H5 and H7 subtypes. The three H9N2 viruses in our study possessed the second connecting peptide motif (R–S–S–R). This finding suggests that these three H9N2 viruses might be capable of becoming highly pathogenic viruses.

After the inoculation of BALB/c mice, we found that the AV1534 virus could replicate well in mouse lungs and cause about 2% weight loss. However, the AV1535 and AV1548 viruses were not able to replicate in the lungs of mice. According to the results of phylogenetic and molecular characterization analysis, we suggested that gene segments and several mutations might contribute to the pathogenicity of these three H9N2 viruses in mice. The PB2, PB1, PA, HA, NA, and M genes of AV1534, AV1535, and AV1548 viruses all originated from the viruses isolated from poultry, but the NS gene of the AV1534 virus was closely related to A/swine/Taizhou/5/2008 (H9N2) isolated from swine in 2008. Several mutations or deletions were also reported relating to the host range and virulence. In our study, all of the three H9N2 viruses had a Q216L mutation, which indicated that these viruses had the characteristics of human influenza virus-like receptor specificity. All of the three H9N2 viruses encoded a 3 aa deletion at the NA stalk region, which has been shown to alter the rates of virus growth, virulence, and transmission in chickens and mammals [35,36,37,38,39,40]. They all had an S31N mutation in the M2 protein, which is associated with amantadine resistance [24]. The NS1 proteins of the three H9N2 viruses were 217 aa in length and lacked the C-terminal amino acids, which contain a PDZ domain ligand (PDZ-L); this suggests that these viruses might increase their virulence to humans [25]. The three H9N2 viruses had the NS1 S42 mutation that plays an important role in the inhibition of IFN production and in the virulence of the virus in mice [26]. Although the three viruses all had the above molecular characteristics, it was worth noting that only the AV1534 virus had an A588I substitution in the PB2 protein, which could enhance virus replication in mammalian cells and virulence in mice [23]. Since the pathogenicity of avian influenza virus is a polygenic trait, other factors may be likely to contribute to the impact on pathogenicity. Our findings highlighted the importance of the NS gene and A588I substitution of the PB2 protein in viral pathogenicity. Further research about how they affect pathogenicity will be needed.

Since 1998, the vaccination strategy has been extensively used to control H9N2 avian influenza in China. Currently, more than fifty different types of vaccines have been used. However, because of the complex breeding and trading patterns of the poultry industry, H9N2 AIV has continuously evolved. In our study, we demonstrated that the three H9N2 AIVs were all multiple genotype reassortants, with genes originating from Y280-like, F/98-like, and G1-like viruses. The pathogenicity of these viruses to chickens has increased, and these viruses are a threat to mammals. Our research is of great significance to prevent and control H9N2 avian influenza.

## Figures and Tables

**Table 1:** Influenza viruses with the highest nucleotide homology to each gene of three H9N2 viruses, as determined by BLAST search in the GenBank.

Strains	Gene ^a^	Closest Viruses in GenBank	Homology (%)
A/chicken/Shandong/AV1534/2011(H9N2)	HA	A/Chicken/Tianjin/2009(H9N2)	97.9
	NA	A/Chicken/Tianjin/2009(H9N2)	99.8
	PB2	A/chicken/Shandong/Li-2/2010(H9N2)	98.7
	PB1	A/chicken/Shandong/01/2009(H9N2)	98.9
	PA	A/chicken/Henan/13/2008(H9N2)	98.8
	NP	A/chicken/Shandong/zc2/2009(H9N2)	99.3
	M	A/chicken/Shandong/02/2008(H9N2)	99.9
	NS	A/swine/Taizhou/5/2008(H9N2)	99.5
A/chicken/Shandong/AV1535/2011(H9N2)	HA	A/chicken/Anhui/LJT/2010(H9N2)	99.1
	NA	A/chicken/Beijing/HD/2010(H9N2)	98.3
	PB2	A/chicken/Anhui/HF/2010(H9N2)	98.9
	PB1	A/Duck/Fujian/1753/2009(H9N2)	99.2
	PA	A/chicken/Shanghai/96/2009(H9N2)	98.5
	NP	A/Duck/Fujian/1753/2009(H9N2)	98.9
	M	A/chicken/Shandong/10/2010(H9N2)	99.3
	NS	A/chicken/Jiangsu/Q3/2010(H9N2)	99.4
A/chicken/Shandong/AV1548/2011(H9N2)	HA	A/chicken/Shandong/H/2009(H9N2)	99.6
	NA	A/chicken/Shandong/HL/2010(H9N2)	99.4
	PB2	A/chicken/Anhui/HF/2010(H9N2)	99.1
	PB1	A/chicken/Hunan/2237/2010(H9N2)	99.1
	PA	A/chicken/Guangdong/1683/2009(H9N2)	99.1
	NP	A/chicken/Shandong/LY/2008(H9N2)	98.6
	M	A/chicken/Beijing/HD/2010(H9N2) A/chicken/Shanghai/96/2009(H9N2)	99.6
	NS	A/chicken/Jiangsu/Q3/2010(H9N2)	99.5

^a^ PB, polymerase basic subunit; PA, polymerase acidic subunit; HA, hemagglutinin; NP, nucleoprotein; NA, neuraminidase; M, matrix; NS, nonstructural.

**Table 2:** Replication in the chickens of the three H9N2 virus after being inoculated intranasally ^a^.

Strains	Virus Replication on Three Days Post-Inoculation (DPI) (log_10_EID_50_/0.1 mL) ^b^				
	Lung	Trachea	Spleen	Kidney	Brain
AV1534	2.58 ± 1.88	2.17 ± 1.16	1.67 ± 0.14	- ^c^	1.92 ± 0.52
AV1535	4.75 ± 1.30	3.50 ± 0	2.83 ± 0.58	2.25 ± 0.66	2.25 ± 0.66
AV1548	5.42 ± 0.14	3.50 ± 0	2.17 ± 1.16	2.83 ± 0.95	1.75 ± 0.43

^a^ Six-week-old specific-pathogen-free (SPF) chickens were inoculated intranasally (i.n.) with 10^6^EID_50_ of the AV1534, AV1535, and AV1548 viruses in a volume of 0.2 mL; three chickens were chosen for virus titer on 3 DPI in each group, and the lungs, trachea, spleen, kidneys, and brain of the three chosen chickens were collected for virus titer in eggs. ^b^ A value of 1.5 was assigned if the virus was not detected from the undiluted sample in three embryonated chicken eggs. Virus titers are expressed as means ± standard deviation in log_10_EID_50_/0.1 mL of tissue. ^c^ Not detected.

**Table 3:** Virus titers in oropharyngeal and cloacal swabs from chickens.

viruses		Virus Shedding and Mean Infectivity Titer on the Days Post-Inoculation (log_10_EID_50_/0.1 mL ± SD ^a^)													
		1 Day		3 Days		5 Days		7 Days		9 Days		11 Days		13 Days	
		O ^b^	C ^c^	O	C	O	C	O	C	O	C	O	C	O	C
AV1534	infected	4.13 ± 0.41 (6/6)	1.83 ± 0.44 (3/6)	4.33 ± 0.13 (6/6)	2.71 ± 0.70 (6/6)	4.42 ± 0.14 (3/3)	2.08 ± 0.52 (2/3)	2.75 ± 0.50 (3/3)	1.83 ± 0.38 (2/3)	ND ^d^ (0/3)	ND (0/3)	ND (0/3)	ND (0/3)	ND (0/3)	ND (0/3)
	contact	3.08 ± 0.29 (3/3)	ND (0/3)	3.67 ± 0.14 (3/3)	1.83 ± 0.58 (1/3)	4.17 ± 0.38 (3/3)	2.25 ± 0.43 (3/3)	1.83 ± 0.58 (1/3)	ND (0/3)	ND (0/3)	ND (0/3)	ND (0/3)	ND (0/3)	ND (0/3)	ND (0/3)
AV1535	infected	3.29 ± 0.29 (6/6)	1.63 ± 0.31 (1/6)	3.83 ± 0.59 (6/6)	2.54 ± 0.60 (6/6)	3.33 ± 1.04 (3/3)	2.42 ± 0.88 (2/3)	2.75 ± 0.43 (3/3)	2.83 ± 1.16 (2/3)	ND (0/3)	2.83 ± 1.16 (2/3)	ND (0/3)	2.83 ± 1.16 (2/3)	ND (0/3)	2.67 ± 1.01 (2/3)
	contact	2.92 ± 0.58 (3/3)	192 ± 0.72 (1/3)	3.25 ± 0.75 (3/3)	1.75 ± 0 (3/3)	2.25 ± 1.09 (2/3)	2.83 ± 0.95 (3/3)	2.42 ± 0.14 (3/3)	2.42 ± 1.01 (2/3)	ND (0/3)	2.17 ± 1.16 (1/3)	ND (0/3)	2.17 ± 1.16 (1/3)	ND (0/3)	1.58 ± 0.14 (1/3)
AV1548	infected	3.67 ± 0.34 (6/6)	1.58 ± 0.13 (2/6)	4.42 ± 0.13 (6/6)	2.25 ± 0.67 (4/6)	4.50 ± 0 (3/3)	2.17 ± 0.58 (2/3)	2.42 ± 0.88 (3/3)	2.33 ± 0.88 (2/3)	ND (0/3)	1.83 ± 0.58 (1/3)	ND (0/3)	1.75 ± 0.43 (1/3)	ND (0/3)	ND (0/3)
	contact	3.50 ± 0 (3/3)	1.92 ± 0.72 (1/3)	3.92 ± 0.52 (3/3)	2.17 ± 0.58 (2/3)	4.08 ± 0.29 (3/3)	2.50 ± 0 (3/3)	3.42 ± 0.14 (3/3)	2.33 ± 0.14 (3/3)	2.17 ± 0.58 (2/3)	ND (0/3)	1.75 ± 0.43 (1/3)	ND (0/3)	ND (0/3)	ND (0/3)

^a^ A value of 1.5 was assigned for calculations if the virus was not detected from the undiluted sample in three embryonated chicken eggs. Virus titers are expressed as mean ± standard deviation in log_10_EID_50_/0.1 mL of tissue. ^b^ Oropharyngeal swabs. ^c^ Cloacal swabs. ^d^ Not detected.
